# Case report: passive transfer of hepatitis B antibodies from intravenous immunoglobulin

**DOI:** 10.1186/1471-2334-14-99

**Published:** 2014-02-22

**Authors:** Simon Parker, Eliza Gil, Patricia Hewitt, Katherine Ward, Yasmin Reyal, Sasha Wilson, Jessica Manson

**Affiliations:** 1 Flat 19, 3 St Pancras Way, London NW1 0PB, UK; 2 Department of Rheumatology, University College London Hospitals, 3rd Floor, 250 Euston Road, London NW1 2PG, UK; 3 Division of Infection & Immunity, University College London, Room 133, 74 Huntley Street, London WC1E 6AU, UK; 4 Haematology and Blood Transfusion, University College London, 60 Whitfield Street, London W1T 4EU, UK; 5 Transfusion Microbiology Office, NHSBT Colindale, Charcot Road, Colindale, London NW9 5BG, UK

**Keywords:** Hepatitis B virus, Intravenous immunoglobulin, Passive antibody transfer, Seronegative inflammatory polyarthritis, Lymphoma, Haematopoietic stem cell transplant, Immunosuppression

## Abstract

**Background:**

Prior to initiating immunosuppressive therapy in the treatment of autoimmune inflammatory conditions, it is a requirement to screen for certain viral serology, including hepatitis B (HBV). A positive result may indicate the need for antiviral therapy, or contraindicate immunosuppression all together. An accurate interpretation of serological markers is therefore imperative in order to treat patients appropriately. We present a case of passive anti-HBV antibody transfer following intravenous immunoglobulin (IVIg) infusion, in which misinterpretation of serology results almost led to inappropriate treatment with antiviral therapy and the withholding of immunosuppressive agents. This phenomenon has been previously reported, but awareness remains limited.

**Case presentation:**

A 50 year old Caucasian gentleman with a history of allogeneic haematopoietic stem cell transplant for transformed follicular lymphoma was admitted to hospital with recurrent respiratory tract infections. Investigation found him to be hypogammaglobulinaemic, and he was thus given 1 g/kg of intravenous immunoglobulin. The patient also disclosed a 3-week history of painful, swollen joints, leading to a diagnosis of seronegative inflammatory polyarthritis. Prior to initiating long term immunosuppression, viral screening found hepatitis B serology suggestive of past infection, with positive results for both anti-HBc and anti-HBs antibody, but negative HBV DNA. In response, prednisolone was weaned and the local hepatology team recommended commencement of lamivudine. Having been unable to identify a source of infection, the case was reported to the local blood centre, who tested a remaining vial from the same batch of IVIg and found it to be anti-HBc and anti-HBs positive. Fortunately the blood products were identified and tested prior to the patient initiating HBV treatment, and the effect of a delay in starting disease-modifying therapy was inconsequential in light of an excellent response to first-line therapies.

**Conclusion:**

Misinterpretation of serology results following IVIg infusion may lead to significant patient harm, including unnecessary antiviral administration, the withholding of treatments, and psychosocial damage. This is especially pertinent at a time when we have an ever increasing number of patients being treated with IVIg for a wide array of immune-mediated disease. Passive antibody transfer should be considered wherever unexpected serological changes are identified.

## Background

Reactivation of hepatitis B virus (HBV) is a recognised complication of immunosuppressive therapy, potentially resulting in hepatocellular injury, liver failure and death [1]. As such, the European Association for the Study of the Liver (EASL) recommend that all patients receiving cytotoxic or immunosuppressive therapy are screened for serological markers of HBV infection (HBsAg, anti-HBc, and anti-HBs) (Table 1) [2]. The majority of cases of reactivation occur in patients with chronic (HBsAg positive, HBV DNA >2000 IU/ml) or inactive (HBsAg positive, HBV DNA <2000 IU/ml) HBV infection. In these instances prophylactic antiviral therapy is advised [3,4]. There are also increasing numbers of cases of HBV reactivation among patients with occult HBV infection, where HBsAg is negative and anti-HBc positive, particularly where immunosuppression is with rituximab [5,6]. The management of these patients remains controversial [7]. The lack of available data leads to variation in management and a cautious approach may be advisable [2].

**Table 1:** Hepatitis B serology profiles

**Status**	**HBsAg**	**HBsAb**	**HBcAB IgM**	**HBcAB IgG**	**HBeAg**	**HBeAb**	**HBV-DNA**
Acute infection	+	-	+	-	+	-	+
Resolved acute infection	-	+	-	+	-	+	-
Chronic carrier	+	-	-	+/-	-	+	-
Chronic active infection	+	-	-	+/-	+	-	+
Vaccinated	-	+	-	-	-	-	-
Passive Ab transfer	-	+/-	+/-	+/-	-	+/-	-

Given the clinical implications, the correct interpretation of hepatitis B viral serology in the context of immunosuppression is of great importance. We describe a case of passive anti-HBV antibody transfer from an intravenous immunoglobulin infusion which almost resulted in unnecessary treatment for HBV infection, and urge clinicians to consider passive antibody transfer as a cause of positive viral serology.

## Case report

A 50 year old gentleman with a history of allogeneic haemopoietic stem cell transplant for transformed follicular lymphoma was admitted to hospital in July 2012 with pneumonia. He had undergone reduced intensity transplant in January 2008, with modest red cell and platelet support, and had a biopsy-proven high grade relapse in March 2009, treated with rituximab followed by a donor lymphocyte infusion. He had been in metabolic complete remission since then, and had been well, though with occasional respiratory tract infections. In early 2012 these became more frequent. He was found to be hypogammaglobulinemic, presumed to be secondary to rituximab, and was given 1 g/kg of intravenous immunoglobulin (Vigam®).

**Figure 1:**
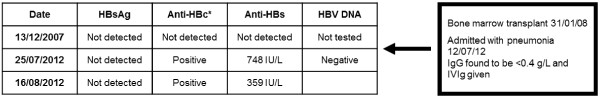
Timeline of events and serology results.*No absolute titre provided.

The patient also disclosed a 3 week history of painful, swollen joints, as well as morning stiffness of 5-6 hours for the several months, primarily affecting the small joints of the hands, right knee and both ankles. He was diagnosed with a seronegative inflammatory polyarthritis, and initially treated in hospital with steroid injection to the right knee, a short course of oral prednisilone and commencement of hydroxychloroquine. He responded well, and following discharge the decision was made to continue long term hydroxychloroquine and to initiate sulfasalazine. Prior to the initiation of this immunosuppressive therapy, routine viral serology was requested including hepatitis B, hepatitis C and HIV. The hepatitis B serology was reported as indicative of past infection, with positive results for both anti-HBc and anti-HBs antibody, but no detectable HBsAg or HBV DNA (Figure 1). His ALT, bilirubin and albumin were in the normal range (22 iU/ml, 6 mcmol/ml and 42 g/L respectively), and his alkaline phosphatase was slightly elevated at 143 IU/L (normal range 40-129 IU/L), but normalized with control of his arthritis and was 107 IU/L the following month.

The abnormal hepatitis B serology was unexpected, as prior to transplant, serology and HBV DNA testing of both donor and recipient were negative, and his sibling donor underwent repeat negative testing when donor lymphocytes were harvested post-transplant. In addition, he had been intensively followed up during the period of profound immunosuppression post-transplant and then the phase of immune reconstitution, with no clinical evidence of a hepatitic illness and normal liver function tests.

In response to these results his prednisolone was weaned and a referral made to the local hepatology team, who recommended commencement of lamivudine, to be continued for the duration of his immunosuppressive therapy.

Discussion of our findings with the patient failed to indicate a probable source of the infection. He denied any illicit drug use or unsafe sexual practice, and the possibility of a sexually transmitted infection led to considerable marital discord. It was also curious that an immunosuppressed patient could contract acute HBV without clinical signs or symptoms, any derangement to his liver function tests, and without the development of a chronic infection.

The case was reported to the local blood centre for consideration of investigation of the 2008 blood components as the source of the HBV infection. At that time, the possibility of the positive HBV markers resulting from the IVIg treatment he received for hypogammaglobulinaemia a few weeks previously was also raised. The patient had received 10 × 10 g vials of Vigam® IVIg (manufacturer: BPL; BioProducts Laboratory ltd) and batch details had been recorded in medical records. A vial remaining from the same batch was tested and found to be both anti-HBc and anti-HBs positive, suggesting the patient could have been the passive recipient of anti-HBc and anti-HBs antibody. This suggestion was supported by ongoing blood monitoring which demonstrated a fall in anti-HBs titre, from 748 mIU/ml initially, to 359 mlU/ml 3 weeks later.

Fortunately the blood products were identified and tested prior to the patient initiating HBV treatment with lamivudine, and the effect of a delay in starting DMARD therapy was inconsequential in light of an excellent response to first-line therapies.

## Discussion and conclusions

IVIg is a plasma-derived product consisting of concentrated immunoglobulin, predominantly IgG. It was first introduced for the treatment of primary humoral immunodeficiencies, but has since been used in the treatment of a variety of immune-mediated diseases, such as acquired hypogammaglobulinemia, idiopathic thrombocytopenia and HIV-associated thrombocytopenia, for example [8]. The passive transfer of antibody from IVIg has previously been reported, [9-11] but there remains limited awareness of this, and passive antibody transfer is not routinely considered in the interpretation of a recipients viral serology results. This may be in part due to the assumption that all blood products are screened for viral antibody and discarded if positive. It is true that this process does occur in some blood services, but this does not necessarily apply to all plasma suppliers, even within the same country. More commonly, donors are screened for HBsAg and HBV DNA only, allowing contributions from non-infectious patients with positive HBV serology to be used [12]. Vigam® is produced by BPL, a UK company, but manufactured from non-UK plasma. The fact that the final product contained anti-HBc demonstrates that the starting plasma was not screened for anti-HBc.

A 2010 Canadian case-control study looked retrospectively for an association between anti-HBc seropositivity and past exposure to IVIg, and found an odds ratio of 16 for anti-HBc positive participants having had past IVIg infusions (95% confidence interval, 1.5-166.1). Furthermore, samples of IVIG from 3 of 5 different blood product manufacturers tested positive for anti-HBc [13]. The manufacturer’s Summary of Product Characteristics (SPC) for the product used in this case clearly states that: “Vigam® Liquid contains mainly immunoglobulin G (IgG), and has a broad spectrum of antibodies against various infectious agents. Vigam® Liquid contains the IgG antibodies present in the normal population and is prepared from pooled plasma from not fewer than 1000 donors”. IVIg has a 21- to 24-day half-life, meaning that antibodies passively acquired will be cleared within 3-4 months of the final dose, [11] and this case clearly demonstrates the falling antibody titres seen in passive antibody transfer.

The risk of infectious complications from IVIg is extremely low, with stringent donor screening requirements meaning there have been no reported cases of HBV or HIV transmission [8]. The importance of passive transfer of antibodies as a common consequence of the administration of IVIg relates to the risk of misinterpretation of serology results, and hence mismanagement of patients, rather than an infection risk. This risk is also present with other immunoglobulin-based products, such as subcutaneous immunoglobulin (SCIg) or specific Ig preparations, which contain high concentrations of antibody to a specific pathogen (in such instances the risk may be reduced, however, due to the relative reduction of other antibodies). An incorrect diagnosis of occult HBV infection may not only lead to the unnecessary prescription of potentially harmful antiviral agents but also the patient being denied beneficial treatments. In this case, the patient was advised to start lamivudine, a nucleoside analogue most suitable for the treatment of those with low (<2000 IU/ml) HBV DNA levels [2]. Although having a relatively benign toxicity profile, known potential side effects of lamivudine include elevations in amylase, lipase and creatine kinase, nausea, headache, dizziness, and pancreatitis [14]. There is also the potential for significant psychosocial harm as a result of an incorrect diagnosis. Chronic HBV infection and carrier states can have a significant negative impact on health-related quality of life, resulting in significant psychiatric morbidity (predominantly depression) [15,16].

With an ever increasing number of patients being treated with IVIg for a wide array of immune-mediated disease, it is important that passive antibody transfer is considered when unexpected serological changes are identified. Our case helps to demonstrate some of the clues that may lead the clinician to recognise a false-positive result:

1) A patient history inconsistent with infection, i.e. lack of source, symptoms or signs.

2) Recent transfusion of immunoglobulin-containing products.

3) The presence of antibodies without viral antigen or DNA.

4) The presence of IgG anti-HBcAb but lack of IgM anti-HBcAb antibody (in our case, we tested for IgG only. Given the predominance of IgG in IVIg products, it would have been prudent to check IgM levels as well, as the likely negative result would have further pointed towards a false-positive conclusion).

5) Falling antibody titres over the following weeks.

Increased awareness and active consideration of the passive transfer of clinically significant antibodies from immunoglobulin treatments will help prevent unnecessary anxiety, antiviral therapy and the withholding of essential immunosuppressive agents. Patients should be informed, appropriately counselled and reassured as to the effect that this treatment may have on subsequent blood tests, and where possible, pre-treatment serological screening of both the patient and the blood product should be considered.

### Consent

Written informed consent was obtained from the patient for publication of this Case report and any accompanying images. A copy of the written consent is available for review by the Editor of this journal.

## Competing interests

The authors declare that they have no competing interests.

## Authors’ contributions

SP was the main author of the manuscript. EG provided intellectual input and revised the manuscript. KW was involved in the clinical care of the patient, provided intellectual input and revised the manuscript. YR and SW were involved in investigating the case and blood sample testing. PH investigated the case, provided intellectual input and revised the manuscript. JM was the consultant in charge of the case, provided intellectual input and revised the manuscript. All authors read and approved the final manuscript.

## Authors’ information

Simon Parker: Foundation Year 1 Doctor; Eliza Gil: Core Medical Trainee; Jessica Manson: Consultant Rheumatologist; Katherine Ward: Consultant Virologist; Yasmin Reyal: Transplant Registrar; Sasha Wilson: Lead Transfusion Practitioner; Patricia Hewitt: Consultant in Transfusion Medicine/Clinical Transfusion Microbiology.

## Pre-publication history

The pre-publication history for this paper can be accessed here:

http://www.biomedcentral.com/1471-2334/14/99/prepub
